# Population‐based age‐specific estimates of dementia in Canada

**DOI:** 10.1002/alz.089492

**Published:** 2025-01-09

**Authors:** Lindsay J Collin, Cathy Lally, W Dana Flanders, Nancy N Maserejian, Cai Gillis

**Affiliations:** ^1^ Epidemiologic Research & Methods, LLC, Atlanta, GA USA; ^2^ Huntsman Cancer Institute, University of Utah, Salt Lake City, UT USA; ^3^ Emory University, Rollins School of Public Health, Atlanta, GA USA; ^4^ Biogen, Cambridge, MA USA

## Abstract

**Background:**

Dementia is a heterogeneous syndrome caused by diseases that impact cerebral functioning leading to progressive cognitive decline and loss of functional independence. Approximately 55 million people worldwide are living with dementia; however, population‐based estimates of dementia are challenging due to non‐standardized surveillance in most countries. We estimated the number of dementia cases among Canadian residents in 2022.

**Method:**

To create age‐specific estimates of dementia in Canada, we compiled data from Statistics Canada. These data included the 2021 Canadian Census (to estimate the total population and type of residence—institutionalized vs. non‐institutionalized), the 2019/2020 Canadian Community Health Survey (to estimate the number of non‐institutionalized persons with dementia), and the 2013 Survey of Neurological Conditions in Institutions in Canada (to estimate the number of institutionalized persons with dementia). These data were used to compute estimates of the number of persons with dementia by age group (65‐69, 70‐74, 75‐79, 80‐84, 85‐89, 90+) in Canada in 2022. Using the overall Canadian population estimates for these age groups, we then calculated age‐specific estimates of dementia per 100,000 persons.

**Result:**

In 2022, approximately 364,280 individuals 65+ years in Canada were living with dementia and the prevalence increased by age. Among those 65‐69 years the estimated prevalence of dementia was 1,343 per 100,000 persons and increased to 25,350 per 100,000 persons among those 90+ years. The prevalence of dementia in non‐institutionalized Canadians 65+ years was approximately 2.1%, and the prevalence among institutionalized Canadians was 52.5%. Of those with dementia, 41% were non‐institutionalized and 59% institutionalized. The proportion of non‐institutionalized dementia cases exceeded the institutionalized cases among those younger than 80 years (65% vs. 35%); however, for those 80+ years, there was a higher proportion of institutionalized cases compared with non‐institutionalized cases (71% vs. 29%).

**Conclusion:**

Dementia is a substantial source of morbidity in Canada, with increasing prevalence in an aging population. Standardized surveillance is necessary to understand the needs of this growing patient population, which would lead to more reliable estimates and allow for proper allocation of resources to improve care.

